# Enhanced efficiency fertilizers, potato production, and nitrate leaching in the Wisconsin Central Sands

**DOI:** 10.1002/jeq2.20672

**Published:** 2025-01-16

**Authors:** Tracy Campbell, Matthew Ruark, Edward Boswell, Birl Lowery

**Affiliations:** ^1^ Department of Soil Science University of Wisconsin–Madison Madison Wisconsin USA

## Abstract

Maintaining yield goals while reducing nitrate‐nitrogen (NO_3_‐N) leaching to groundwater is a challenge for potato (*Solanum tuberosum*) production in the Wisconsin Central Sands as well as across the United States. The objectives of this study were to quantify the effect of conventional and enhanced efficiency nitrogen (N) fertilizers on NO_3_‐N leaching, crop yield, and N uptake in potatoes. We compared five N treatments, which include a 0 N control and 280 kg ha^−1^ as ammonium sulfate and ammonium nitrate (AS/AN), polymer‐coated urea (PCU), urea with a urease inhibitor (Urea+UI), or urea with a UI and a nitrification inhibitor (Urea+UI+NI). The study occurred on grower fields during the 2009, 2010, and 2011 growing seasons, and NO_3_‐N leaching was measured with equilibrium tension lysimeters. PCU resulted in a reduction in NO_3_‐N leaching and an increase in yield compared to AS/AN in a year with large early‐season rainfall; Urea+UI also reduced NO_3_‐N leaching in this year. In 2010, large plot‐to‐plot variation and 250 kg ha^−1^ of additional N applied by the grower masked our ability to see differences among fertilized treatments. In 2011, a year with less intense rainfall events, no differences among treatments were observed. Collectively, these results show a potential benefit to PCU, but these benefits are only realized under specific seasonal weather conditions. Overall, the percentage of applied N lost to leaching during the growing season and removed in biomass was relatively low, suggesting substantial amounts of NO_3_‐N leaching outside of the growing season.

## Core Ideas

- Polymer‐coated urea (PCU) fertilizer reduced NO_3_‐N leaching when large rainfall events were concentrated early in the season.
- Enhanced efficiency fertilizers did not consistently reduce NO_3_‐N leaching for all study years.
- PCU had no negative impacts on potato yield across all years.
- NO_3_‐N leaching exceeded 80 kg ha^−1^ during two sampling events in the study.
- Nitrogen budget approaches indicate large amounts of unaccounted‐for N in potato cropping systems.

**Abbreviations**
AN: ammonium nitrate
AS: ammonium sulfate
FWMC: flow weighted mean concentration
NI: nitrification inhibitor
PCU: polymer‐coated urea
UI: urease inhibitor
WCS: Wisconsin Central Sands

## 1 INTRODUCTION

Across the US Midwest, agricultural regions are challenged with maintaining profitable crop yields while simultaneously reducing the amount of nitrogen lost to the environment. The Wisconsin Central Sands (WCS), a region characterized by its sandy soil, high hydraulic conductivity, and shallow depth to groundwater, exemplifies many of these challenges. The WCS is the primary potato‐producing region in the state, and this crop requires large amounts of nitrogen (N) fertilizer and frequent irrigation to reduce drought stress and produce large high‐quality yields. As a result, groundwater nitrate‐nitrogen (NO_3_‐N) concentrations can exceed drinking water standards with 20%–25% of local domestic drinking water wells exceeding 10 mg L^−1^ s (Masarik et al., 2018).

Split application of N fertilizer for potato production is standard practice. Previous studies have demonstrated a reduction in NO_3_‐N leaching when fertilizer is applied in split applications with limited impact on potato yield (Kelling et al., 2015; Vos, 1999; Waddell et al., 2000). However, even with split N applications, large rainfall events can still lead to excessive NO_3_‐N losses in sandy soils, especially later in the growing season (Sexton et al., 1996). Polymer‐coated urea (PCU) can be used as an alternative or supplement to split applications to reduce NO_3_‐N leaching losses. The release of N from PCU fertilizers is controlled by soil temperature and moisture (Gandeza et al., 1991; Golden et al., 2011), which slows the release of available N to match crop N needs more closely. Studies conducted to evaluate PCU on potatoes found increased yields over traditional fertilizers (Pack et al., 2006; Wilson et al., 2009; Zvomuya & Rosen, 2001). Several studies in Minnesota demonstrated the added benefit of reducing NO_3_‐N leaching in potato cropping systems using PCU over traditional fertilizers in sandy soils (Wilson et al., 2010; Zvomuya et al., 2003). However, Clément et al. (2020) found PCU led to similar or greater NO_3_‐N losses compared to split applications of conventional fertilizers, concluding that PCU may only be beneficial if substantial rainfall occurs within a few weeks after planting.

Nitrification inhibitors (NIs) can also improve the synchrony of fertilizer N availability and crop N uptake by killing or inhibiting the bacteria Nitrosomonas, which limits the conversion of ammonium to NO_3_‐N (Ruark et al., 2018; Trenkel, 2010), reducing the time NO_3_‐N is susceptible to loss. A global meta‐analysis across a diverse range of crops found that while NIs increased crop productivity and nitrogen use efficiency in relation to the control, the level of success varied considerably but was greatest for crops receiving high amounts of nitrogen fertilizer and grown on coarse‐textured soil (Abalos et al., 2014). However, previous research on potatoes in Wisconsin found that use of NIs reduced harvested US No. 1 tubers in 4 out of 6 site years when used with ammonium‐based fertilizer (Kelling et al., 2011), limiting its feasibility as a best management practice for this crop.

Since there is an extra cost associated with enhanced efficiency fertilizers, evaluation of both agronomic and environmental impacts needs to be executed on farmer fields, across multiple growing seasons, and using quantitative approaches to inform farmer decisions. Most studies quantifying NO_3_‐N leaching losses under potato production have used suction cup samplers (e.g., Clément et al., 2020; Wilson et al., 2010; Zvomuya et al., 2003). While this approach has advantages for comparative studies in terms of ease of installation and sampling, it does require a water budget approach to determine fluxes such as NO_3_‐N leaching. Equilibrium tension lysimeters are recognized as a more accurate method for flux quantification, as they maintain natural drainage patterns with little disturbance to the soil profile (Brye et al., 1999). The objective of the study was to quantify the effect of conventional and enhanced efficiency N fertilizers on NO_3_‐N leaching, crop yield, and N uptake for potatoes in sandy soil. To do so, this research was conducted on farmer fields in the WCS and used equilibrium tension lysimeters to quantify seasonal NO_3_‐N leaching losses.

**Core Ideas**

- Polymer‐coated urea (PCU) fertilizer reduced NO_3_‐N leaching when large rainfall events were concentrated early in the season.
- Enhanced efficiency fertilizers did not consistently reduce NO_3_‐N leaching for all study years.
- PCU had no negative impacts on potato yield across all years.
- NO_3_‐N leaching exceeded 80 kg ha^−1^ during two sampling events in the study.
- Nitrogen budget approaches indicate large amounts of unaccounted‐for N in potato cropping systems.

## 2 MATERIALS AND METHODS

### 2.1 Study sites

This study was conducted in 2009, 2010, and 2011 in a different commercial potato field each year. Two fields (2009 and 2010) were operated by the same grower near Grand Marsh, WI (48°54′34″ N, 89°40′39″ W), and one field (2011) was near Coloma, WI (44°01′21″ N, 89°35′51″ W). The soil in all fields was classified as Plainfield loamy sands (mixed, mesic Typic Udipsamments). This soil was formed from glacial till overlain by glacial outwash representing the bed of glacial Lake Wisconsin, and it is generally composed of deep, well‐drained sands with massive structure and rapid or very rapid saturated hydraulic conductivity (Bockheim & Hartemink, 2017; Hart & Lowery, 1996).

### 2.2 Experimental design

The study was designed as a randomized complete block design replicated three times with five N fertilizer treatments. Plot sizes were 3.66 by 6.10 m, encompassing four potato rows. The N fertilizer treatments consisted of: (i) no experimental N fertilizer applied (0 N), (ii) ammonium sulfate–ammonium nitrate (AS/AN) representing a conventional N fertilizer, (iii) urea with a urease inhibitor N‐(n‐butyl)‐thiophosphoric triamide (Agrotain, Agrotain International LLC) (Urea+UI), (iv) urea impregnated with N‐(n‐butyl)‐thiophosphoric triamide and dicyandiamide (SuperU, Agrotain International LLC) (Urea+UI+NI), and (v) a PCU (ESN, Agrium Advanced Technologies, Agrium, Inc.).

A‐grade Russet Burbank (*Solanum tuberosum* L.) potatoes were cut in halves or thirds and mechanically planted by the growers at 0.9 m row spacing with a seed density of 36,600 seeds ha^−1^. The potatoes were planted on April 17, 2009; April 15, 2010; and May 1, 2011. For the PCU treatment, nitrogen fertilizer was applied once at a rate of 280 kg ha^−1^ between 26 and 34 days after planting in 2009, 2010, and 2011. For the AS/AN, Urea+UI, and Urea+UI+NI treatments, nitrogen fertilizer was applied in split applications, with the first application occurring on May 18, May 19, and May 26 in 2009, 2010, and 2011, respectively; the second fertilizer application occurred on June 3, June 6, and June 17 in 2009, 2010, and 2011, respectively. Fertilizer was incorporated into the soil through hilling, except for the second application in 2011. All treatments, including the 0 N unfertilized treatments, received 32, 39, and 0 kg N ha^−1^ of (NH_4_)_2_SO_4_ at planting in 2009, 2010, and 2011, respectively. During the 2009 and 2010 sampling season, the cooperating growers applied 113 and 250 additional kg ha^−1^ of N through fertigation. As a result, the total N applied in the fertilized treatments was 425, 569, and 280 kg·ha^−1^ of N in 2009, 2010, and 2011; and 145, 289, and 0 kg·ha^−1^ N were applied in the unfertilized treatments in 2009, 2010, and 2011, respectively. A surfactant consisting of 10% alkoxylated polyols and 7% glucoethers (Irrigaid, Aquatrols) was applied at emergence and at the time of tuber initiation fertilization in all 3 years. Aside from the experimental N applications, growers followed their own crop management plans for soil preparation, irrigation, pesticide application, and non‐N fertilization for the duration of the three growing seasons.

### 2.3 Pan lysimeters

In each of the 15 experimental plots, leachate was collected using custom‐fabricated equilibrium tension pan lysimeter as described by Brye et al. (1999). The lysimeters measured 25.4 cm wide by 76.2 cm long by 15.2 cm tall, providing a capacity of at least 120 mm of drainage water, and included a porous stainless‐steel top, a sampling line, and a vacuum line. Lysimeters were installed at a depth of 1 m below potato hilltop on May 11, 2009, April 27, 2010, and May 19, 2011, prior to plant emergence. Holes were hand dug, and soil was repacked by hand on top of and around the lysimeter after installation. Care was taken to place soil back at the depths at which it was removed (i.e., a horizon soil was placed back at the soil surface). The lysimeters were interconnected, and the entire system was connected to a 1/12 hp. vacuum pump (model 900‐13‐58, Thomas) that maintained a constant and continuous suction consistent with 100 cm of water, equivalent to the field moisture capacity of the study soils (Hart & Lowery, 1996).

Drainage water was collected under vacuum from the outflow tube of each lysimeter approximately every 7 days from May 27 through September 24. in each of the 3 years, hereafter referred to as “sampling season.” At each sampling event, the total drainage volume within each lysimeter was measured, and a subsample was collected and placed into a 500‐mL plastic Nalgene bottle. Samples were transported to the laboratory in an ice‐filled cooler, filtered through a 0.45 µm filter within 24 h after collection, and stored at 4°C until analysis.

Drainage water samples were analyzed for NO_3_‐N by the single vanadium chloride, microplate method described in Doane and Howarth (2003), with each sample ran in triplicate. Colorimetric analysis and absorbances were read by a Bio‐Tek Powerwave (BioTek Instruments Inc.) microplate reader. The only exception was that samples from May 28, 2009, were analyzed for NO_3_‐N using an ion chromatograph (Dionex DX500). A subset of samples was re‐analyzed after the project was completed due to extremely high values; these samples were analyzed using a SEAL AQ2 automated discrete analyzer (SEAL Analytical Inc.). In all cases, where the NO_3_‐N concentration was below that of 0.5 mg L^−1^ (the lowest point of the standard curve), a value of 0.25 mg L^−1^ was assigned. Drainage was converted from volume to area by dividing by the area of lysimeter. NO_3_‐N leaching for each sample collection was calculated as the product of NO_3_‐N concentration and drainage. All events in a sampling season were summed to determine sampling season cumulative leaching. Sampling season flow weighted mean concentrations (FWMC) were calculated by dividing the seasonal leaching by the total season drainage.

At each farm, precipitation was measured using a tipping bucket rain gauge (model RG3, Onset Computer Corp.) connected to a datalogger (Model 10X, Campbell Scientific). However, in 2009, National Oceanic and Atomospheric Administration precipitation records recorded at Hancock Agricultural Research Station, approximately 30 km from the study site, were used instead due to data inconsistencies in the tipping bucket method. Irrigation amounts were reported by the growers.

### 2.4 Plant analysis

Tubers from the two center rows of each plot were mechanically harvested on September 15, 2009; August 30, 2010; and September 23, 2012. The tubers were graded into US No. 1, undersize (not retained on a 4.8‐cm screen), and cull (off‐shape, green, diseased, or blemished). The US No. 1 tubers were electronically graded into size categories. Ten potatoes from the 170 to 283 g size class were collected and analyzed for specific gravity with a PW‐2050 (Weltech International Ltd) analyzer and calculated using weight in air divided by weight in water. Total N in tubers was determined using dry combustion with a Leco CNS‐2000 analyzer (Leco Corp.). Tuber dry matter was calculated as a function of specific gravity (Kellock, 1995; Henderson, 2000). Total N uptake in tubers was calculated by multiplying tuber dry matter by tuber N content and by tuber yield.

### 2.5 Calculations and statistics

Two N budget calculations were conducted. First, the potentially leachable N was calculated as the difference between total N applied and N removed in the tuber. Second, the unaccounted‐for N (remaining N) was calculated as the difference between total N applied and the sum of N removed in biomass and the measured NO_3_‐N leaching.

To compare nitrogen fertilizer treatment effects on season totals, data from each year was analyzed independently. We used a linear mixed effects model followed by analysis of variance for cumulative sampling season measurements of NO_3_‐N leaching, FWMC, crop yield, N removed in tuber, potentially leachable N, and unaccounted‐for N; fertilizer treatment was treated as a fixed effect and block was treated as a random effect. Because of a lack of homogeneity in variance for FWMC and NO_3_‐N leaching values between treatments, data were log transformed for the 2010 sampling season. Following analysis of variances, differences in treatment means were determined using Tukey's post hoc analysis means comparison with a *p*‐value of < 0.1 considered significant.

To determine the impact of nitrogen treatment on NO_3_‐N leaching across sampling events, we used a mixed‐effects linear model with fertilizer treatment and sampling event and the interaction between fertilizer treatment and sampling event serving as fixed effects and block as a random effect. Each year was analyzed independently and log transformed due to non‐normality. All analysis was completed in RStudio version 4.2.2 (Posit team, 2023) using the lme4 (Bates et al., 2015) and emmeans (Length, 2023) packages.

## 3 RESULTS

### 3.1 Drainage, irrigation, and rainfall

In total, there were 15, 14, and 14 sampling events during the 2009, 2010, and 2011 sampling seasons, respectively. Average seasonal drainage (across all plots) was 274, 287, and 167 mm for 2009, 2010, and 2011, respectively; this led to water recovery efficiencies [drainage/(irrigation + rainfall)] of 0.32, 0.37, and 0.30. In 2009, the highest amount of drainage was measured during the June 9, 2009, sampling event, with 55 mm across plots recorded. In the week leading up to the June 9 sampling event, 46 mm of rainfall occurred over 3 days. During 2010, the greatest amounts of drainage were measured on July 21 and July 28, 2010, which each followed a week receiving >75 mm of rainfall. The largest amount of drainage from a single sampling event during the 2011 sampling season was lower than the previous 2 years; the largest drainage event, when averaged across plots, was 35 mm recorded on both June 24 and July 22, 2011. The 2009 sampling season received 297 mm of rainfall and 549 mm of irrigation; in 2010, 607 mm of rainfall occurred and 155 mm of irrigation, and the 2011 sampling season measured 267 mm rainfall and 278 mm irrigation. Based on the timing and intensity of the rainfall events, and the differences in management by all three cooperating growers, each year can be considered a separate study that reflects: (i) rainfall‐induced leaching occurring early in the growing season and with additional N applied by the grower (2009), (ii) rainfall‐induced leaching occurring in the middle of the growing season and with additional N applied by the grower (2010), and (iii) a low rainfall intensity season with no additional N applied by the grower (2011). Each year was analyzed separately and considered under the context of these environmental and management scenarios.

### 3.2 Leaching events

Across all five N fertilizer treatments and across all sampling events, NO_3_‐N concentration per sampling event ranged from 0.16 mg L^−1^ to 192 mg L^−1^, with a mean value of 40.7 mg L^−1^. For control treatments (0 N), the average N concentration per sampling event ranged from 0.25 to 63.4 mg L^−1^ across the 3 years of the study. Mean NO_3_‐N concentrations across sampling events exhibited a general trend in all 3 years with increased concentrations coincident with the first large rainfall events and a subsequent declining trend as the season progressed. Similar to NO_3_‐N concentrations per sampling events, average NO_3_‐N leaching per event (averaged across all treatments and plots) also ranged widely from 0.01 to 91.7 kg ha^−1^.

**FIGURE 1:**
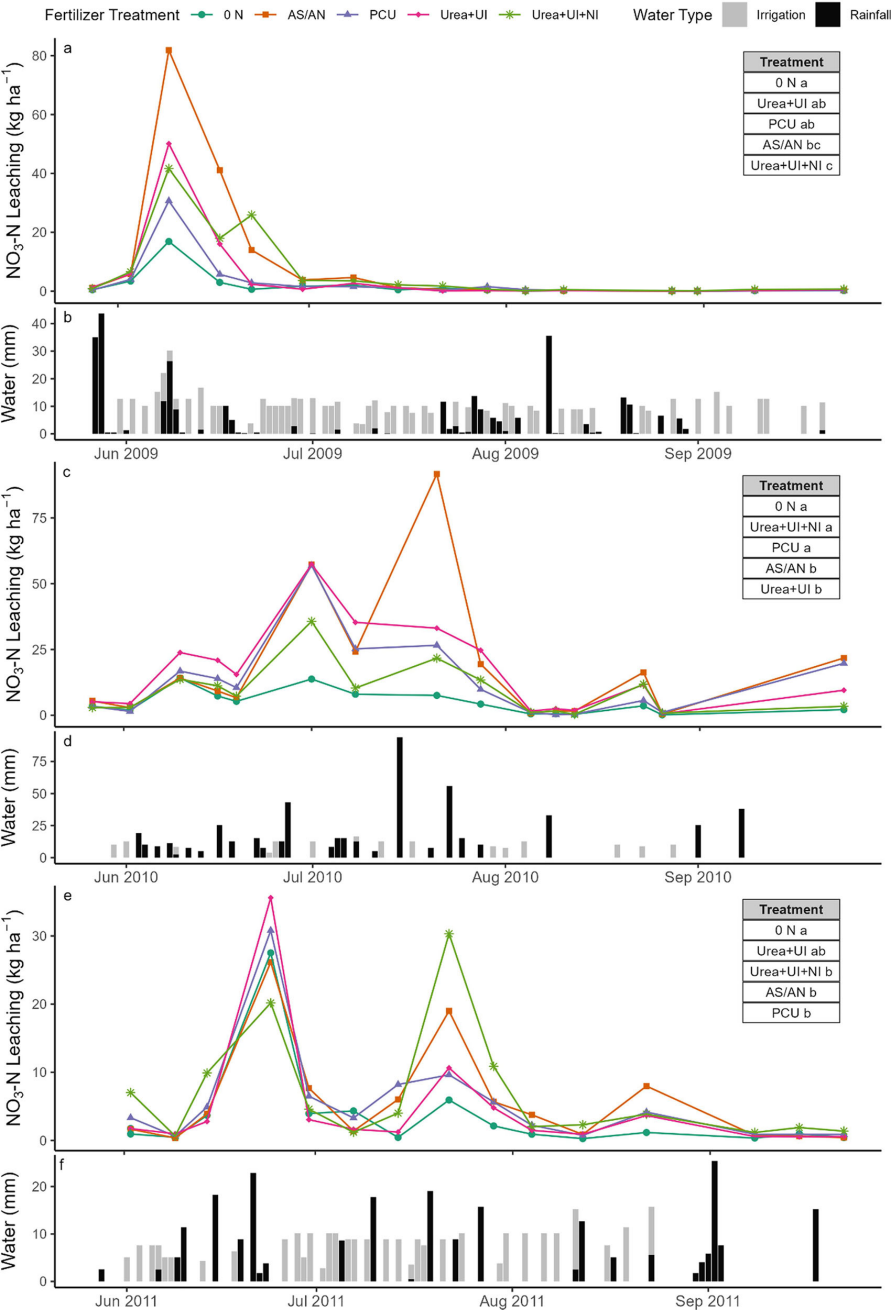
NO_3_‐N leaching across five fertilizer treatments (a, c, and e) and irrigation and rainfall patterns (b, d, and f) for the 2009 (a–b), 2010 (c–d), and 2011 (e–f) sampling seasons. Treatment differences for nitrate leaching, averaged across sampling events, are provided in the inset table, different letters indicate significant differences at *α* = 0.1 (Tukey HSD) and are listed from smallest to largest average value. AS/AN, ammonium sulfate/ammonium nitrate; NI, nitrification inhibitor; PCU, polymer‐coated urea; UI, urease inhibitor.

Based on our linear mixed effects model, which was analyzed separately by year, there were significant effects of fertilizer treatment and sampling time each year, but there was no significant interaction between the treatments. Despite no significant interaction between sampling event and fertilizer treatment, the observed differences between fertilizer treatments within specific sampling events are still of interest, as large leaching events occurring during specific sampling events likely influenced our cumulative results. During the 2009 sampling season, most NO_3_‐N leaching occurred during the first month of the growing season, which coincided with a large rainfall event also occurring during the early growing season (Figure 1a,b). Most notably, on June 9, 2009, the largest NO_3_‐N leaching of the year was measured. The AS/AN treatment leached 81.9 kg ha^−1^ of NO_3_‐N, which was more than four times greater than the 0 N (16.9 kg ha^−1^) treatment and three times greater than PCU (30.7 kg ha^−1^) treatment (Figure 1a). Additionally, all fertilized treatments except for the PCU treatment lost more NO_3_‐N than the 0 N control treatment, with the Urea+UI and Urea+UI+NI leaching 50.1 and 41.7 kg ha^−1^ of NO_3_‐N, respectively (Figure 1a). The peak in NO_3_‐N leaching measured during the June 9, 2009, sampling event occurred 6 days after a second application of fertilizer (for the fertilized, non‐PCU treatments) and followed 38 mm of rainfall (Figure 1b). A similar trend in NO_3_‐N leaching across the fertilizer treatments was also observed during the June 17, 2009, sampling event. During this sampling period, 41.1, 17.9, 16.0, 5.7, and 3.0 kg ha^−1^ of NO_3_‐N were leached under the AS/AN, Urea+UI, Urea+UI+NI, PCU, and 0 N fertilizer treatments, respectively, with the AS/AN treatment leaching more than seven times that of the PCU (Figure 1a).

On average, event NO_3_‐N losses were greater throughout the 2010 sampling season compared to 2009 because of the continued fertilizer application by the cooperating farmer in addition to higher amounts of rainfall throughout the sampling season. After a week of extreme rainfall (94 mm), 91.7 kg ha^−1^ of NO_3_‐N was leached during the July 21, 2010, sampling period under the AS/AN treatment, compared to only 7.54 kg ha^−1^ lost under the 0 N treatment (Figure 1c,d). Under conditions vulnerable to NO_3_‐N leaching, as observed on July 21, 2010, the PCU and inhibitor treatments leached approximately 60%–75% less NO_3_‐N in comparison to the conventional AS/AN approach. However, the large estimated NO_3_‐N leached under the AS/AN was due in part to the wide variability in drainage collected across the three plots, which measured 100, 49, and 41 mm, as well as the variability in NO_3_‐N concentration, which measured 120, 228, and 92 mg L^−1^. Additionally, a sampling event earlier in the growing season (July 1, 2010), did not result in large differences among fertilized N treatments, although they had 2.6–4.2 times greater amounts of leaching compared to the control (Figure 1c).

During the 2011 sampling season, considerably less NO_3_‐N was applied, and considerably less NO_3_‐N was lost during peak leaching events in comparison to the previous sampling seasons. While sampling season rainfall quantities were comparable in 2009 and 2011, in contrast to the 2009 sampling season, the largest NO_3_‐N leaching event was not observed until the end of June, on June 24, 2011 (Figure 1e). This sampling event occurred 6 days after a fertilizer application event and included two rainfall events ranging between 18 and 22 mm and resulted in 35.6, 30.8, 27.5, and 20.17 kg ha^−1^ of NO_3_‐N leached under the Urea+UI, PCU, 0 N, AS/AN, and Urea+UI+NI fertilizer treatments, respectively (Figure 1e,f). In contrast, a different pattern in leaching was observed on July 22, where 30.3, 19.0, 10.6, 9.6, and 5.9 kg ha^−1^ of NO_3_‐N leached under the Urea+UI+NI, AS/AN, Urea+UI, PCU, and 0 N treatments, respectively (Figure 1e).

### 3.3 Sampling season NO_3_‐N leaching and FWMC

**FIGURE 2:**
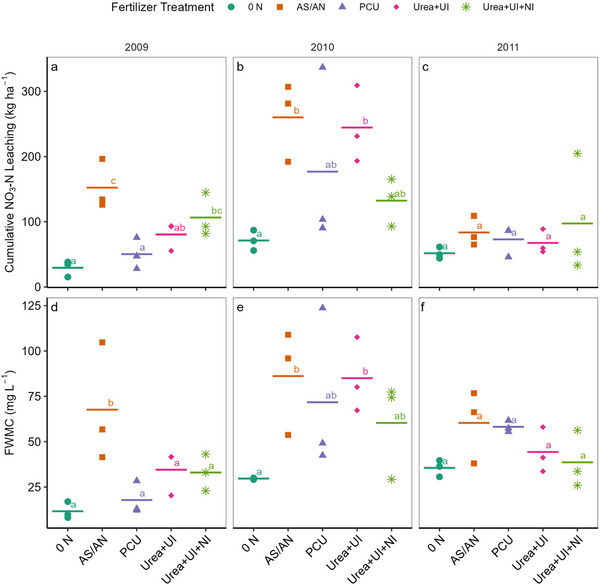
Mean cumulative seasonal NO_3_‐N leaching (kg ha^−1^) (a–c) and mean cumulative seasonal flow weighted mean concentration (FWMC) (mg L^−1^) (d–f) represented by line and plot level averages represented by individual points across five nitrogen fertilizer treatments and three sampling seasons, 2009 (a and d), 2010 (b and e), and 2011 (c and f). Different letters indicate statistically significant differences in seasonal mean NO_3_‐N leaching (kg ha^−1^) or FWMC (mg L^−1^) at *α* = 0.1 (Tukey HSD). AS/AN, ammonium sulfate/ammonium nitrate; NI, nitrification inhibitor; PCU, polymer‐coated urea; UI, urease inhibitor.

There was a significant effect of treatment on NO_3_‐N leaching and FWMC concentrations in 2009 and 2010, but not in 2011. In 2009, during sampling season, NO_3_‐N leaching was significantly affected by nitrogen fertilizer treatment and was approximately fivefold greater in the AS/AN treatment as compared to the 0 N treatment (Table 1; Figure 2). Sampling season NO_3_‐N leaching in the Urea+UI+NI treatment did not differ significantly from the AS/AN treatment. PCU had the lowest total mean NO_3_‐N leaching of the added nitrogen treatments. In 2009, the AS/AN had the greatest FWMC values, and all other N treatments were not different from the 0 N treatment (Table 1; Figure 2). In 2010, despite the wide range in seasonal NO_3_‐N leaching across treatments, only the AS/AN and Urea+UI treatments leached significantly more NO_3_‐N than the 0 N treatment. The same effects were determined for FWMC in 2010. We were unable to determine differences in growing season NO_3_‐N leaching and FWMC in 2011, despite there being a nearly twofold difference among treatments (Table 1; Figure 2).

**TABLE 1:** Measurements of NO_3_‐N leaching (kg ha^−1^), flow weighted mean concentration (FWMC) (mg L−1), irrigation (mm), rainfall (mm) and drainage (mm) for five nitrogen fertilizer treatments across 2009 (549 mm irrigation and 297 mm rainfall), 2010 (155 mm irrigation and 607 mm rainfall), and 2011 (278 mm irrigation and 267 mm rainfall) sampling seasons.

Treatment	NO_3_‐N leaching (kg ha^−1^)	FWMC (mg L^−1^)	Irrigation (mm)	Rainfall (mm)	Drainage (mm)
**2009**					
0 N	29.5a	11.6a	549	297	256
AS/AN	152c	67.6b	549	297	243
PCU	50.4a	17.8a	549	297	288
Urea+UI	80.6ab	34.5a	549	297	240
Urea+UI+NI	107bc	33.0a	549	297	330
**2010**					
0 N	71.3a	29.6a	155	607	227
AS/AN	260b	86.3b	155	607	265
PCU	177ab	71.8ab	155	607	213
Urea+UI	245b	85.0b	155	607	262
Urea+UI+NI	132ab	60.3ab	155	607	223
**2011**					
0 N	518	35.5	278	267	148
AS/AN	83.7	60.3	278	267	143
PCU	73.0	58.2	278	267	127
Urea+UI	67.6	44.3	278	267	154
Urea+UI+NI	97.5	38.6	278	267	218

*Note*: Different letters indicate statistically significant differences (*α* = 0.1).

Abbreviations: AS/AN, ammonium sulfate/ammonium nitrate; NI, nitrification inhibitor; PCU, polymer‐coated urea; UI, urease inhibitor.

### 3.4 Tuber yield and quality

In 2009, there was a significant effect of N treatment on total and US No. 1 yields, with the only difference being between PCU and 0 N (Table 2). No yield effects were determined in 2010 or 2011. In 2009, tuber specific gravity was significantly higher under the 0 N and Urea+UI+NI treatments, and no differences were found in 2010 and 2011. The PCU treatment had greater N content compared to the urea treatments and the 0 N in 2009, and the PCU and AS/AN treatment had greater N content compared to the urea treatments and the 0 N in 2010 (Table 2); no differences between treatments were observed in 2011.

**TABLE 2:** Agronomic yield measured by total yield (Mg ha^−1^), US No. 1 (Mg ha^−1^), and percent of US No. 1 > 170 g (%), specific gravity, and tuber N (%) grown across five nitrogen fertilizer treatments for the 2009, 2010, and 2011 sampling seasons.

Treatment	Total yield (Mg ha−1)	US No. 1 (Mg ha−1)	US No. 1 > 170 g (%)	Specific gravity	Tuber N (%)
**2009**					
0 N	63.3a	55.6a	50.7	1.092b	1.26a
AS/AN	75.7ab	66.3ab	57.0	1.084a	1.46ab
PCU	78.5b	68.3b	59.3	1.084a	1.55b
Urea+UI	68.7ab	61.3ab	48.7	1.085a	1.30a
Urea+UI+NI	67.0ab	57.3a	42.7	1.089b	1.30a
**2010**					
0 N	46.1	31.0	49.0	1.067	1.21a
AS/AN	39.1	25.1	38.7	1.067	1.52b
PCU	49.1	33.7	47.7	1.068	1.56b
Urea+UI	39.4	23.8	33.3	1.068	1.29a
Urea+UI+NI	35.2	23.9	42.0	1.065	1.31a
**2011**					
0 N	34.5	30.0	26.0	1.073	1.34
AS/AN	43.3	39.2	57.3	1.074	1.43
PCU	40.1	37.1	41.7	1.076	1.45
Urea+UI	39.4	34.8	55.3	1.077	1.38
Urea+UI+NI	42.8	38.8	39.3	1.077	1.40

*Note*: Different letters indicate statistically significant differences (*α* = 0.1).

Abbreviations: AS/AN, ammonium sulfate/ammonium nitrate; NI, nitrification inhibitor; PCU, polymer‐coated urea; UI, urease inhibitor.

### 3.5 Nitrogen budget

The potentially leachable N, calculated as the difference between N applied and N removed in tuber, was generally much greater than the actual amount of NO_3_‐N leached. Notable exceptions were in 2009, where the sampling season NO_3_‐N leaching in AS/AN represented 84% of the potentially leachable N (Table 3). In contrast, the sampling season NO_3_‐N leaching from the PCU treatment only represented 31% of the potentially leachable N. When averaged across all N treatments, the sampling season N represented 44% and 50% of the potentially leachable N in 2010 and 2011, respectively (Table 3). The amount of N remaining reflects the amount that is unaccounted for in our known quantities of the budget (i.e., input, leaching, and removal), which can represent the combination of N remaining in the system, unaccounted for inputs, and unaccounted N losses. In 2009, where an additional 145 kg ha^−1^ of N was applied by the grower, the AS/AN treatment only had an N unaccounted value of 29.2 kg ha^−1^, suggesting we captured most of the N applied in our measurements (Table 3). In contrast, the other N treatments had much more N left unaccounted for (>100 kg ha^−1^). The unaccounted‐for N in 2010 was two to three times greater than those in 2009, and values in 2011 were similar to or slightly less than those in 2009 (Table 3). When the unaccounted‐for N was calculated as a percent of the total N applied, most values ranged between 25% and 43%.

**TABLE 3:** Nitrogen budget estimates are based off the amount of N fertilizer applied (kg ha^−1^), the potential leachable N, the amount of N lost through leaching (kg ha^−1^), and the amount of N removed in tuber biomass (kg ha^−1^) across five nitrogen fertilizer treatments.

Treatment	N applied (kg ha^−1^)	Potential leachable N (kg ha^−1^)	NO_3_‐N leaching		N removed		Unaccounted‐for N	
			kg ha^−1^	%	kg ha^−1^	%	kg ha^−1^	%
**2009**								
0 N	145	−41.5a	29.5a	20.3	187b	129	−71.0c	−49.0
AS/AN	425	191bc	161c	38.0	235ab	55.2	29.2b	6.9
PCU	425	160b	50.4a	11.9	265a	62.3	110ab	25.8
Urea+UI	425	228c	80.6ab	19.0	197b	46.3	148a	34.7
Urea+UI+NI	425	225c	107bc	25.1	200b	47.0	119ab	27.9
**2010**								
0 N	289	184a	71.3a	24.7	105	36.3	113b	38.9
AS/AN	569	457b	260b	45.7	112	19.7	197ab	34.6
PCU	569	424b	177ab	31.1	145	25.5	247ab	43.4
Urea+UI	569	473b	245b	43.0	96.2	16.9	228ab	40.1
Urea+UI+NI	569	484b	132	23.3	84.2	14.8	352a	62.0
**2011**								
0 N	0	−91 a	51.8	NA	91.2	NA	−143b	NA
AS/AN	280	157 b	83.7	29.9	124	44.1	72.8a	26.0
PCU	280	161 b	73.0	26.1	119	42.6	87.8a	31.3
Urea+UI	280	169 b	67.6	24.1	111	39.6	101a	36.2
Urea+UI+NI	280	156 b	97.5	34.8	124	44.2	58.7a	21.0

*Note*: N applied as fertilizer was considered the only input and NO_3_‐N leached and N removed from biomass were considered the only outputs. The percentage of NO_3_‐N leached, N removed, and N unaccounted for were determined relative to the amount of nitrogen fertilizer applied. Different letters indicate statistically significant differences (*α* = 0.1).

Abbreviations: AS/AN, ammonium sulfate/ammonium nitrate; NA, not applicable; NI, nitrification inhibitor; PCU, polymer‐coated urea; UI, urease inhibitor.

## 4 DISCUSSION

### 4.1 Enhanced efficiency fertilizers

PCU was most effective at reducing NO_3_‐N leaching when large rainfall events were isolated to the early portion of the growing season. In 2009, the PCU treatment resulted in a total NO_3_‐N leaching comparable to the 0 N treatment and threefold lower than the split application of AS/AN. The 2009 growing season is characterized by large rainfall events occurring 10 days after PCU application. In contrast, the 2011 growing season N fertilizer application rates were similar to University of Wisconsin‐Madison Extension recommendations (Laboski et al., 2012), but PCU did not result in less NO_3_‐N leaching due to leaching inducing rainfall events occurring later in the growing season. This suggests that there is a capacity for PCU to reduce NO_3_‐N leaching, but only early in the growing season, at times when the N is still mostly protected in the polymer coating. In 2010, under conditions of high N applications (twice the recommended rate), there were also no differences between PCU and other fertilizer treatments; use of PCU with overapplications of N did not lead to any reductions in NO_3_‐N leaching. Previous researchers have reported reductions in NO_3_‐N leaching in potato production systems with PCU compared to split applications of other fertilizer sources (Wilson et al., 2010; Zvomuya et al., 2003). While others, such as Clément et al. (2020), report no difference in NO_3_‐N leaching between PCU and other N fertilizers. These findings suggest that the benefit of PCU fertilizers to water quality may only become apparent under excess irrigation and rainfall during certain points of the growing season (Bero et al., 2014), in combination with moderately high nitrogen fertilizer inputs (Quemada et al., 2013).

Potato yields in the PCU treatment were the highest of all fertilizer treatments and the only treatment to result in significantly greater yields in comparison to the 0 N treatment in 2009. However, these findings were inconsistent across years, and there was no yield benefit associated with PCU fertilizer during the 2010 and 2011 growing seasons. Past research conducted under comparable conditions similarly found little difference in potato yield across conventional and PCU treatments (Bero et al., 2014; Cambouris et al., 2016). In contrast, others have reported the use of control release fertilizers to increase crop yields (Hopkins et al., 2008; Liegel & Walsh, 1976; Pack et al., 2006; Zvomuya et al., 2003), which help support our 2009 findings. The discrepancy in the relationship between potato yield and PCU fertilizer across field seasons may be partially explained by differences in rainfall, irrigation, and the amount of nitrogen fertilizer applied in each year of the study. More specifically, past research has proposed the idea that PCU may have a greater beneficial impact on crop yield when N application rates are below the recommended amount (Bero et al., 2014). Previous work in the region on corn cropping systems emphasizes the ability of rainfall patterns to hinder the effectiveness of PCU fertilizer. Rui et al. (2019) found that though PCU fertilizer tended to be both agronomically and environmentally advantageous over other fertilizer treatments, the effectiveness of PCU was reduced under high rainfall years.

While PCU reduced NO_3_‐N leaching compared to the conventional AS/AN approach under certain environmental and management conditions with no cost to yield, our results also indicate that the PCU treatment left more N unaccounted and available for leaching outside of the growing season. Recent studies conducted on similar soils support this finding, demonstrating that certain PCU fertilizers had greater amounts of residual soil N after crop harvest in comparison to conventional split application approaches (Clément et al., 2019; Venterea et al., 2011). As a result, it is likely that the PCU treatment would see continued NO_3_‐N leaching throughout the fall and spring (Clément et al., 2021). Past research in the same region as our study found that 54% of NO_3_‐N leaching in potato cropping systems occurs outside of the growing season (Masarik, 2023), when leaching can be exacerbated due to high rainfall and low evaporative transpiration (Shrestha et al., 2010). Moving forward, longer monitoring of NO_3_‐N leaching is needed to fully capture impacts of nitrogen fertilizer on NO_3_‐N leaching inside and outside of the potato growing season.

The impact of nitrification and urease inhibitors on NO_3_‐N leaching and crop yield was inconsistent across years. In 2009, the treatment incorporating both a nitrification and urease inhibitor (Urea+UI+NI) leached significantly more NO_3_‐N than the 0 N treatment. However, the treatment incorporating only a urease inhibitor (Urea+UI) was not statistically different from the 0 N control. In contrast, in 2010 the Urea+UI+NI treatment was not significantly different than the control, while the Urea+UI treatment leached substantially more NO_3_‐N than the 0 N treatment. In 2011 there was no statistically significant difference in NO_3_‐N leaching across any of the treatments. A meta‐analysis conducted by Quemada et al. (2013) evaluating the effectiveness of NIs on NO_3_‐N leaching across a range of cropping systems also found the effectiveness to vary considerably, reducing NO_3_‐N leaching between 5% and 30%. Similar to NO_3_‐N leaching, there was little discernible difference in crop yield with the use of inhibitors across all 3 years of the study, though in 2009, the Urea+UI+NI treatment did result in significantly less yield than the PCU treatment. Kelling et al. (2011) also found inconsistent impacts on crop yield, with 3 site years resulting in increased crop yield associated with the use of inhibitors, and 4 site years associated with a decrease in crop yield.

### 4.2 Nitrogen budgets

NO_3_‐N leaching under potato production can be quite substantial regardless of the fertilizer used. Previous studies in this region have quantified annual NO_3_‐N leaching under potato at 224 kg ha^−1^ (Kraft & Stites, 2003). Our growing season‐only leaching amounts ranged from 22% to 72% of that estimation across the 2009 and 2011 growing season. While total leaching may be similar, conventional fertilizers still pose the largest risk of leaching from a single event. The amount of NO_3_‐N leached during a single sampling time exceeded 75 kg ha^−1^ twice in the AS/AN treatment, representing a significant portion of the applied N. Arriaga et al. (2009) also showed the potential for large NO_3_‐N losses in this region, with peak losses nearing 50 kg ha^−1^ day^−1^.

Overapplication of N on potato in the WCS will have disproportionately large influences on NO_3_‐N concentrations in groundwater. As part of our on‐farm research, growers applied additional N through fertigation and were unable to prevent this application from occurring on our research plots. In 2010, the collaborating grower applied an additional 250 kg ha^−1^ of N through fertigation, leading to a total amount of N applied that was double that of recommended guidelines. As a result, the NO_3_‐N leaching amounts were double (or more) than those in other years. These extra N applications reflect the lack of economic disincentive to over‐application of N. Often, especially for high‐value crops such as potato, the cost of applying excess nitrogen fertilizer to reduce the risk of crop yield loss outweighs the additional cost of fertilizer (Mitchell, 2004; Mitchell et al., 2021) without considering the impacts to the environment and human health.

During the 2009 and 2011 growing seasons, when the 0 N treatments received 145 kg ha^−1^ and 0 kg ha^−1^, respectively, more N was removed from the system either as NO_3_‐N leaching or potato biomass than the amount of nitrogen fertilizer applied. Previous research also found more N removed than applied for unfertilized potato, with tuber N removal ranging from 63 to 73 kg ha^−1^ of N (Errebhi et al., 1998), slightly lower than our estimated 91 kg ha^−1^ NO_3_‐N in 2011. Saffigna and Keeney (1977) also observed a mismatch in the nitrogen added and nitrogen removed from the system based on a similar N budget approach. These findings can be explained by unaccounted‐for N inputs. The NO_3_‐N in irrigation water can provide a substantial amount of NO_3_‐N to the crop if the NO_3_‐N concentration in irrigation water is high and if the crops are well irrigated (Delaune & Trostle, 2012; Cahn et al., 2017; Campbell et al., 2023). Based on recent work in the WCS by Campbell et al. (2023), we estimated 92, 27, and 50 kg ha^−1^ of NO_3_‐N may have been applied through irrigation water in 2009, 2010, and 2011, respectively, only partially explaining the unaccounted‐for N in 2011. While residual soil N is usually low in sandy soil, it is possible that soil N supplied the crop with unaccounted N. Alternatively, we may have underestimated the decomposition of previous plant residues, such as soybean residue from the previous crop. Past research in the region has indicated the ability for N to rapidly mineralize in the sandy soil (West et al., 2016), which may help account for the unaccounted‐for nitrogen in our N budget calculations. Despite discrepancies in the N budget, the equilibrium tension lysimeters offered an effective tool for capturing N fluxes while maintaining accurate estimates of the water budget.

### 4.3 Limitations

While our findings highlight the benefits of PCU fertilizer over the conventional AS/AN treatment under certain weather and management conditions, high levels of plot‐to‐plot variability in drainage amount and NO_3_‐N leaching may have interfered with our ability to determine statistical differences across fertilizer treatments. In 2009, seasonal drainage values at the plot level ranged from 188 to 406 mm. During the 2010 sampling season, the plot‐to‐plot variability was smaller, falling between 223 and 385 mm. Similar to 2009, the 2011 sampling season resulted in a wide variability in cumulative drainage values across plots, ranging from 75 to 364 mm. Wide variability in drainage of potato cropping systems has been documented previously; Nocco et al. (2018) also found wide variability in recharge under potato cropping systems, which was not explained by variability in soil texture. Additional studies using lysimeters have indicated that because of the hills and furrows in potato cropping systems, there may be greater spatial variability in drainage (Gee et al., 2009; Herath et al., 2014), and similarly, Saffigna et al. (1976) found wide variability in infiltration under potato canopies. While variability is expected when measuring between treatments, we also observed high levels of variability within fertilizer treatments. Most notably, during the 2010 sampling season, the PCU treatment demonstrated high levels of plot‐to‐plot variability, with cumulative NO_3_‐N leaching measuring 342, 119, and 118 kg ha^−1^. Even when standardized based on drainage quantity, the FWMC values were also highly variable under the PCU treatment, with plots measuring 115, 43, and 46 mg L^−1^ during the 2010 sampling season. Previous research conducted by Bero et al. (2014) experienced similar challenges, with high levels of variability impeding clear statistical differences in NO_3_‐N leaching between fertilizer treatments. Additionally, the variability in seasonal rainfall amount and rainfall patterns, as well as the variability in the amount of nitrogen fertilizer applied between study years, added to the inherent variability in our results.

## 5 CONCLUSION

Our results highlight the challenges of managing potato production for yield and quality goals while minimizing groundwater contamination. PCU fertilizer provided clear reductions in NO_3_‐N leaching and increases in potato yield, but only in 1 year of the study. In this year, N was applied moderately above the recommended rate, and leaching inducing rainfall occurred only during the early growing season. Results were less consistent under other environmental and management conditions, and PCU did not reliably outperform the other fertilizer treatments in terms of yield or NO_3_‐N leaching under such conditions. Environmental and management conditions influence the effectiveness of enhanced efficiency fertilizers and should be considered by growers in the decision‐making process, especially considering climate change projections. While PCU fertilizers can reduce NO_3_‐N leaching relative to conventional fertilizers, NO_3_‐N leaching was still substantial, with 50 to 177 kg ha^−1^ leached during the 4‐month sampling season. However, overapplication of nitrogen may be the more pressing management issue, as in two of the 3 years, >400 kg ha^−1^ of NO_3_‐N was applied by growers.

## AUTHOR CONTRIBUTIONS

**Tracy Campbell**: Formal analysis; validation; visualization; writing—original draft; writing—review and editing. **Matthew Ruark**: Conceptualization; data curation; funding acquisition; investigation; methodology; writing—original draft; writing—review and editing. **Edward Boswell**: Formal analysis; validation; visualization; writing—original draft; writing—review and editing. **Birl Lowery**: Conceptualization; data curation; funding acquisition; investigation; methodology; writing—review and editing.

## CONFLICT OF INTEREST STATEMENT

The authors declare no conflict of interest.
